# The perceived importance of words in large font guides learning and selective memory

**DOI:** 10.3758/s13421-024-01555-2

**Published:** 2024-04-19

**Authors:** Dillon H. Murphy, Matthew G. Rhodes, Alan D. Castel

**Affiliations:** 1 Department of Psychology, University of California, Los Angeles, CA 90095 USA; 2 Department of Psychology, Colorado State University, Fort Collins, CO USA

**Keywords:** Memory, Metamemory, Judgments of importance, Value, Font size

## Abstract

People are often presented with large amounts of information to remember, and in many cases, the font size of information may be indicative of its importance (such as headlines or warnings). In the present study, we examined how learners perceive the importance of information in different font sizes and how beliefs about font size influence selective memory. In Experiment 1, participants were presented with to-be-remembered words that were either unrelated or related to a goal (e.g., items for a camping trip) in either small or large font. Participants rated words in large font as more important to remember than words in small font when the words in a list were unrelated but not when the words were schematically related to a goal. In Experiments 2 and 3, we were interested in how learners’ belief that font size is indicative of importance translates to their ability to selectively encode and recall valuable information. Specifically, we presented participants with words in various font sizes, and larger fonts either corresponded to greater point values or smaller point values (values counted towards participants’ scores if recalled). When larger fonts corresponded with greater point values, participants were better able to selectively remember high-value words relative to low-value words. Thus, when to-be-remembered information varies in value, font size may be less diagnostic of an item’s importance (the item’s importance drives memory), and when the value of information is consistent with a learner’s belief, learners can better engage in selective memory.

## Introduction

In everyday life, people are presented with massive amounts of information but are often unable to remember everything. As a result, people are usually selective with their memory and one’s goals may influence how important it is to remember certain information. For example, when going on a camping trip, a tent or sleeping bag may be more crucial to remember than a toothbrush or marshmallows, and these more important items are therefore more likely to be remembered (e.g., McGillivray & Castel, 2017; see also Murphy & Castel, 2020). Thus, the importance of information can guide memory (see Luna et al., 2019), and importance can be inferred based on one’s goals (e.g., important items to bring on a trip) as well as the physical cue properties of information (e.g., the large font size of headlines in a newspaper or a long list of medication side effects presented in a small font). Consequently, people’s memory, as well as their predictions about what they need to remember and what information is important to know for the future, may be sensitive to such cues.

The conceptual link between perceptual factors like font size and the perceived importance or value of information has been the subject of many prior studies. For example, Li et al. (2015) delved into how perceptual cues guide self-regulated learning, showing that features like font size affect how individuals select and prioritize items during learning. Rhodes and Castel (2008) theorized that font size could serve as a cue for importance in memory predictions, a notion echoed by Luna et al. (2019), who provided empirical evidence for this relationship. Specifically, Luna et al. (2019) demonstrated that learners attribute higher predictions of memory performance (as measured by judgments of learning or JOLs) and importance judgments to words presented in larger fonts, and that this could partially mediate the effect of font size on JOLs. Additionally, Alban and Kelley (2013) showed that physical characteristics, such as the perceived weight of study materials, can influence metacognitive judgments. These studies collectively suggest that perceptual cues, beyond their sensory impact, play a role in the cognitive appraisal of the significance of information. However, there remains a need to explore how perceptual cues, when interpreted as indicators of importance, influence the processes of learning and subsequent retrieval.

Previous work indicates that learners better remember information judged as important to remember relative to information judged as less important to remember. For example, Murphy and Castel (2021a) presented participants with pictures of children and their food preferences (including foods they were allergic to). Learners judged the foods the children were allergic to as most important to remember and subsequently best remembered those foods (see also Murphy et al., 2023b). Similarly, Murphy and Castel (2021b) asked participants to remember a list of items to pack for a camping trip, and observed that items judged as most important to remember were best remembered (see also Murphy & Castel, 2022a). Thus, the perceived importance of remembering can guide encoding.

Although value or importance can influence memory (Castel et al., 2002; Elliott et al., 2020; for reviews, see Knowlton & Castel, 2022; Madan, 2017), and learners are generally metacognitively aware of their selective memory (e.g., Murphy et al., 2021), learners often incorrectly believe that certain intrinsic qualities of words can influence memorability (cf. Koriat, 1997). For example, Rhodes and Castel (2008) presented learners with words in a large and small font to remember for a later test. After studying each word, participants made a JOL indicating how likely they were to remember it (see Rhodes, 2016, for a review of JOLs). Results revealed that although there were no significant differences in recall as a function of font size, participants expected to better remember words in a large font (see also Ball et al., 2014; Besken & Mulligan, 2013; Blake & Castel, 2018; Halamish, 2018; Kornell et al., 2011; Luna et al., 2018; Mueller & Dunlosky, 2016; Price et al., 2016). Thus, learners sometimes use font size as a cue to predict memorability.

Recent work has demonstrated that when both font size and inter-item relation are manipulated together, the influence of font size on JOLs is reduced or eliminated (Chang & Brainerd, 2023). This suggests that learners sometimes use font size as a cue to predict memorability, but when items in a list are related, learners attend to different cues when assessing memorability, possibly the importance or value of the information. For example, Murphy et al. (2022) demonstrated that people remembered high-value information even if presented in a small font, indicating that item value may supersede font size. Thus, when participants have other cues to use as a basis for their JOLs, such as value or the semantic relatedness of a word pair, the font size bias can be reduced (Rhodes & Castel, 2008; but see Undorf et al., 2018, for instances of other cues that do not reduce the font size effect).

Previous research on metamemory and font size has consistently shown an effect of font size on JOLs, but a much smaller or negligible effect of font size on actual memory performance (font size generally has minimal effects on memory; Halamish, 2018; Luna et al., 2018; Murphy & Castel, 2022b; Price et al., 2016; Undorf et al., 2018; for a review, see Chang & Brainerd, 2022,1). This discrepancy may reflect the lack of differential processing or cognitive engagement with larger font words compared to smaller font words. For instance, participants likely do apply superior learning strategies for large compared with smaller font words. Consequently, font size has minimal impact on memory performance. However, font size may capture attention and impact learning if font size is related to importance (e.g., headlines, warning labels). Thus, people may believe that font size is a valid cue for importance given prior experiences.

Rating words in a large font as more likely to be remembered than words in a small font likely reflects faulty beliefs about factors that affect memory (e.g., Kornell et al., 2011; Mueller et al., 2014; Undorf & Zimdahl, 2019). Specifically, learners may believe that font size is an impactful predictor of future memorability and incorporate font size into their metamemory judgments, particularly when it is a salient source of variability among otherwise similar items. However, learners may also believe that font size is indicative of importance (e.g., headlines usually appear in large font while footnotes appear in small font; Luna et al., 2019) and importance is predictive of memory (e.g., Murphy & Castel, 2021a, 2021b; Murphy et al., 2023b). Thus, larger fonts may also be interpreted as being more important to remember based on an individual’s prior experiences with larger fonts, thus influencing recall (see Luna et al., 2019). As a result, if learners believe that information in a large font is more important than information in a small font, then using font size to guide memory may be an effective strategy to maximize the value of remembering.

The importance of information is not always made explicit to learners; oftentimes, we must determine the value of remembering on our own. Some recent work has demonstrated that when the value of information is not explicit, people use task experience to learn what information is the most important to remember. For example, Silaj et al. (2023) presented participants with lists of items belonging to different categories (e.g., mammals, birds, fish), and words from certain categories were more valuable than others. Results revealed that even without explicit value cues, participants learned which categories were of greater value, and applied this to future learning trials. Thus, participants used the learned semantic reward structure to guide recall and optimize memory. Applied to font size, if learners perceive words in a large font as more memorable due to their associated value (Luna et al., 2019), they should be better able to selectively remember high-value information when the value of the information aligns with this belief. Specifically, participants should be more selective in their memory when words in a large font are more valuable than words in a small font (consistent with participants’ beliefs) relative to conditions where words in a small font are more valuable (inconsistent with participants’ beliefs).

## The current study

In the current study, we examined how font size is used to guide memory. In Experiment 1, learners judged the importance of to-be-remembered information (either unrelated words or a list of words that could be related to a specific goal) presented in either large or small font. If people judge words in larger fonts as more important to remember, this would indicate that learners likely expect words in a large font to be better remembered because they may be more important (see Luna et al., 2019). In Experiments 2 and 3, learners studied words in various font sizes and the font size was either a positive predictor (i.e., the bigger the word, the more it was worth) or a negative predictor (i.e., the bigger the word, the less it was worth) of the value of each item. If the font size effect (greater expected memory for large words) is driven by learners’ belief that font size is indicative of memory performance, learners should be better able to engage in selective memory when the value of information is consistent with their beliefs (i.e., larger fonts correspond to more valuable words).

## Experiment 1

In Experiment 1, we presented participants with words in either large or small font. After each word was presented, participants were asked to make a judgment of importance (JOI). For some participants, the words in each list were unrelated, but, for other participants, we added a context for each list such that the to-be-remembered words were schematically related to a goal (e.g., items to pack for a vacation) to examine how goals guide JOIs when learners have a basis for making importance ratings. Prior work with these materials has demonstrated that people often remember items that are rated as important (McGillivray & Castel, 2017; Murphy & Castel, 2021b), but it remains unclear how font size may influence memory in this context. We examined this issue using situations in which font size is not necessarily diagnostic of recall or importance (but could influence JOIs and thus subsequent memory) to determine how font size could bias judgments in the absence of being related to importance. Although semantic relatedness in word pairs can influence both JOLs and associative memory, when a goal is presented with single items to consider in terms of importance, we expected that this goal would guide people’s judgments more so than the font size of the presented items. Specifically, while some participants may use font size as a basis for their JOIs (see Luna et al., 2019), this may be greatly reduced or even eliminated by the goal context for each list as participants have an additional subjective basis for their JOIs, beyond their beliefs about font size. As such, an item’s intrinsic importance to a goal (e.g., when packing for a vacation, it is crucial to remember your passport and less important to remember a hat) may greatly reduce or override any effects of font size as participants may use the goal to guide recall to ensure that important items are best remembered (see also Murphy et al., 2022).

### Method

#### Participants

Participants were 98 undergraduate students (*M*_*age*_ = 20.38 years, *SD*_*age*_ = 3.16 years). In each experiment, participants were recruited from the University of California Los Angeles (UCLA) Human Subjects Pool, were tested online, received course credit for their participation, and were excluded from analysis if they admitted to cheating (e.g., writing down answers) in a post-task questionnaire (they were told they would still receive credit if they cheated). This exclusion process resulted in one exclusion. Due to the binary outcome and intricate interactions encompassed in our multilevel models, performing power analyses was not practical (see Scherbaum & Ferreter, 2009, for a review of the difficulties estimating statistical power for cross-level interactions in multilevel modeling). Therefore, we determined our sample sizes based on previous studies with a similar design (e.g., Murphy & Knowlton, 2022), given the expectation of detecting a medium effect size (interactions between font size and value). As such, based on the expectation of detecting a medium effect size, in each experiment, we aimed to collect around 50 participants in each condition. Additionally, each participant was only allowed to participate in one experiment (i.e., all participants in each study were naïve).

#### Materials

Unrelated words were four-letter nouns, and on the log-transformed Hyperspace Analogue to Language frequency scale (with lower values indicating lower frequency in the English language and higher values indicating higher frequency), words ranged from 5.48 to 12.88 (*M* = 9.73, *SD* = 1.48). In terms of concreteness (with lower values indicating lower concreteness and higher values indicating higher concreteness), words ranged from 4.26 to 4.87 (*M* = 4.64, *SD* = .18). When the words were related, each list consisted of 20 items that were consistent with a theme (going camping, going on vacation, items for a child’s party, items to bring to class, ingredients for making lasagna, and going on a picnic; see Appendix Table 2 for stimuli adapted from McGillivray & Castel, 2017) and participants were told to imagine themselves in the scenario. For example, participants were told to imagine that they were going camping and would be presented with items that could be taken on the trip. Words ranged from two to 12 letters (*M* = 6.25, *SD* = 1.87). On the log-transformed Hyperspace Analogue to Language frequency scale, words ranged from 5.22 to 12.37 (*M* = 8.45, *SD* = 1.55). In terms of concreteness, words ranged from 4.14 to 5.00 (*M* = 4.82, *SD* = .17).^2^ Words were classified according to the English Lexicon Project website (Balota et al., 2007).

#### Procedure

Participants were presented with a series of either related (*n* = 53) or unrelated (*n* = 45) to-be-remembered words, with half of the words presented in a large font (48 point) and half presented in a small font (12 point). Each list contained 20 words and included ten large words and ten small words, with words randomly selected to be presented in a large or small font for each participant (i.e., each word could appear in any serial position and in any font size). Words were presented one at a time, for 4 s each. Participants were told that they would be tested on their memory for the presented words but were not given any information regarding word size. Prior to studying each list, participants studying related words were given a brief introduction to the theme of the list (e.g., “For this list, please imagine you are going to go on a camping trip. This list will contain items you need to remember to bring.”).

After each word was presented, participants were asked, “How important is it to remember this word?” Participants answered with a number between 0 and 100, with 0 meaning not important and 100 meaning very important. The judgment phase was self-paced and the word did not appear on the screen while participants made their judgments. Immediately after all 20 words were presented, participants were given a 1-min free-recall test in which they had to recall as many words as they could from the current list; participants could recall words in any order they wished. This process was repeated for a total of six study-test cycles, but words were never repeated (i.e., each list contained new words that had not been previously studied).

### Results

**Fig. 1:**
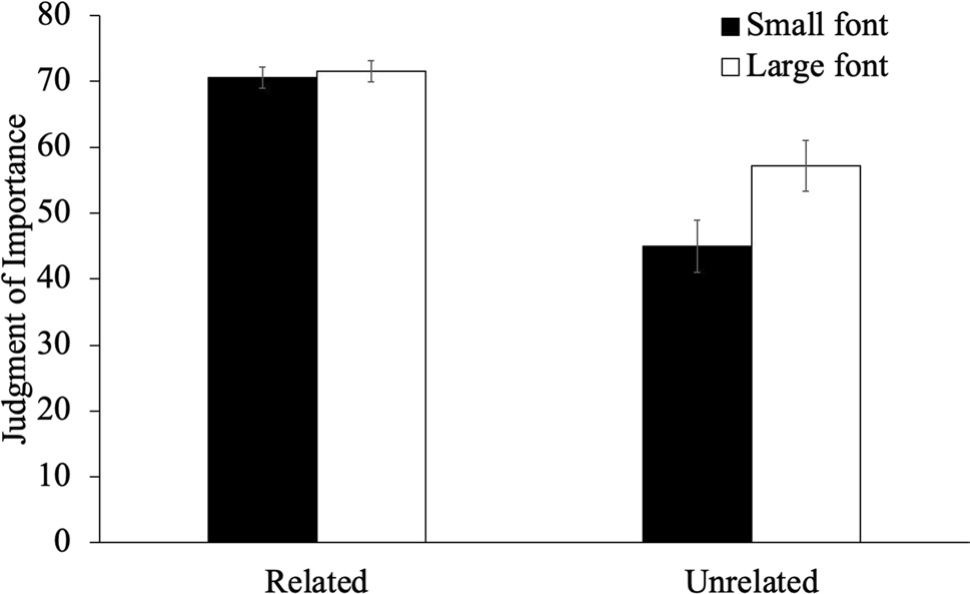
Judgments of importance as a function of font size and list relatedness in Experiment 1. Error bars reflect the standard error of the mean

To compare participants’ JOIs (see Fig. 1), we conducted a 2 (list relatedness: unrelated, schematically related) × 2 (size: small, large) mixed-design ANOVA. Results revealed that large words (*M* = 64.93, *SD* = 20.81) were rated as more important to remember than small words (*M* = 58.82, *SD* = 23.67), [*F*(1, 96) = 16.37, *p* < .001, η_p_^2^ = .15]. Additionally, related words (*M* = 71.06, *SD* = 11.31) were rated as more important to remember than unrelated words (*M* = 51.06, *SD* = 23.75), [*F*(1, 96) = 29.67, *p* < .001, η_p_^2^ = .24]. Critically, font size interacted with list relatedness [*F*(1, 96) = 12.10, *p* < .001, η_p_^2^ = .11], such that, when the words were unrelated, big words (*M* = 57.18, *SD* = 26.11) were rated as more important to remember than small words (*M* = 44.94, *SD* = 26.47), [*t* = 5.12, *p*_holm_ < .001, *d* = .62], but when the words were related, big words (*M* = 71.52, *SD* = 11.59) were rated as similarly important to remember as small words (*M* = 70.60, *SD* = 12.01), [*t* = .42, *p*_holm_ = .677, *d* = .05].

**Fig. 2:**
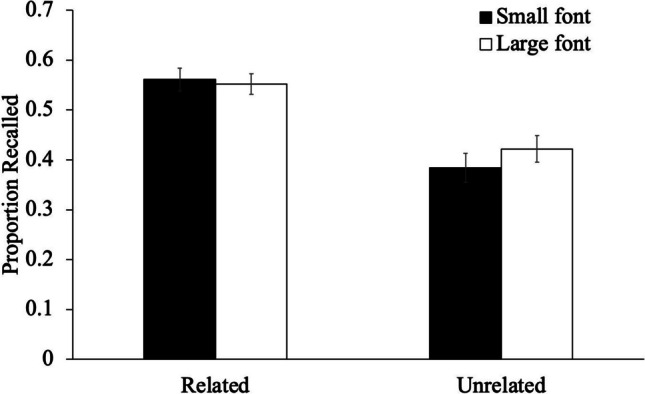
The proportion of words recalled as a function of font size and list relatedness in Experiment 1. Error bars reflect the standard error of the mean

Next, to examine recall (see Fig. 2), we conducted a 2 (list relatedness: unrelated, schematically related) × 2 (size: small, large) mixed-design ANOVA. Results revealed that large words (*M* = .49, *SD* = .18) were similarly likely to be recalled as small words (*M* = .48, *SD* = .20), [*F*(1, 96) = 1.33, *p* = .251, η_p_^2^ = .01]. However, related words (*M* = .56, *SD* = .15) were better recalled than unrelated words (*M* = .40, *SD* = .17), [*F*(1, 96) = 21.99, *p* < .001, η_p_^2^ = .19]. Font size did not interact with list relatedness [*F*(1, 96) = 3.53, *p* = .063, η_p_^2^ = .04].

To determine if participants remembered words they judged as important to remember, we computed a multilevel model (MLM) whereby we treated the data as hierarchical or clustered (i.e., multilevel), with items nested within individual participants. Because recall at the item level was binary (correct or incorrect), we conducted logistic MLMs to examine recall. In these analyses, the regression coefficients are given as logit units (i.e., the log odds of correct recall). We report exponential betas (e^B^) and their 95% confidence intervals, which give the coefficient as an odds ratio (i.e., the odds of correct recall divided by the odds of not recalling a word). Thus, e^B^ can be interpreted as the extent to which the odds of recalling a word changed. Specifically, values greater than 1 represent an increased likelihood of recall while values less than 1 represent a decreased likelihood of recall.

We conducted a logistic MLM with item-level recall modeled as a function of participants’ item-level JOIs, font size, and relatedness. Results revealed that JOIs significantly predicted recall [e^B^ = 1.01, CI: 1.01 – 1.01, *z* = 15.71, *p* < .001]^3^ such that words rated as important were more likely to be recalled than words rated as less important to remember. Font size did not significantly predict recall [e^B^ = 1.02, CI: .94 – 1.11, *z* = .47, *p* = .641], but related words were better recalled than unrelated words [e^B^ = .65, CI: .48 – .88, *z* = -2.76, *p* = .006]. Font size did not interact with list relatedness [e^B^ = .92, CI: .78 – 1.09, *z* = -.93, *p* = .354] or JOIs [e^B^ = 1.00, CI: 1.00 – 1.00, *z* = .53, *p* = .599]. JOIs interacted with list relatedness [e^B^ = 1.00, CI: 1.00 – 1.01, *z* = 2.24, *p* = .025], though an analysis of the simple effects indicates that the predictiveness of JOIs when the words were related [e^B^ = 1.01, CI: 1.01 – 1.01, *z* = 11.53, *p* < .001] was minimally different from when the words were unrelated [e^B^ = 1.01, CI: 1.01 – 1.02, *z* = 11.08, *p* < .001]. There was not a three-way interaction between font size, list relatedness, and whether participants made JOIs [e^B^ = 1.00, CI: 1.00 – 1.01, *z* = .08, *p* = .933].

### Discussion

In Experiment 1, participants studied lists of items related to a goal or unrelated words to remember for a later test. Results revealed that large words were rated as more important to remember than small words, but only when the words were unrelated. Font size did not significantly interact with relatedness when examining participants’ recall, but participants better remembered words they rated as important to remember. Together, Experiment 1 indicates that learners may expect to better remember words presented in a large font (e.g., Rhodes & Castel, 2008) because they perceive words in large font as more important to remember than words in small font (see also Luna et al., 2019). However, when learners have a cue besides font size to guide memory – intrinsic importance in this case – font size becomes less relevant to the learner and importance may be assessed based on the learner’s goals. Thus, an item’s intrinsic importance to a goal (e.g., when making lasagna, it is crucial to remember noodles) may override any effects of font size, consistent with prior work (Murphy et al., 2022).

## Experiment 2

Experiment 1 revealed that when words are unrelated, learners use font size as an indicator of importance. To test the idea that learners have a belief that large words are more valuable than small words, in Experiment 2 we presented participants with lists of unrelated to-be-remembered words in four different font sizes (e.g., 12, 36, 60, 84). Participants were told that the words in each list differed in point value and that they could score points by recalling words on the test. Additionally, we told participants that the size of the font indicates the value of the word (explicit value instructions; the value scheme was not stated in Experiment 3). For some participants, larger words were worth more points, while other participants were told that smaller words were worth more points (all participants had a goal to maximize their score). Participants were told their score after each list and this was repeated for eight study-test cycles to examine whether task experience changes memory for valuable words. Consistent with participants’ beliefs that large words are more important, we expected participants to be more selective when point value positively related to font size.

We also expected this effect to be particularly pronounced on early lists. Specifically, when participants first encounter a task, their existing beliefs and biases should have the strongest influence before experience or feedback can adjust their strategies. Because participants tend to naturally associate larger font size with greater importance (Luna et al., 2019), this belief is likely to guide their initial encoding process, making them more attentive to and better at recalling words presented in a larger font that also carry higher point values. This effect is expected to be most evident during the early stages of the task before participants have had sufficient opportunity to adapt their beliefs based on the task's specific reward structure and feedback from their recall performance. Using multiple study-test cycles is common when using free-recall tasks, especially when assessing value-directed remembering (see Knowlton & Castel, 2022). These cycles provide a greater volume of data and enable researchers to track how memory and recall strategies evolve and become more efficient with repeated exposure (e.g., McGillivray & Castel, 2011). Accordingly, participants in Experiment 2 were given multiple study-test cycles.

### Method

#### Participants

Participants were 109 undergraduate students (*M*_*age*_ = 20.53 years, *SD*_*age*_ = 2.28 years). One participant was excluded for admitting to cheating.

#### Materials

The to-be-remembered items were 128 unrelated words (never repeated) between four and eight letters (*M* = 6.19, *SD* = 1.27). On the log-transformed Hyperspace Analogue to Language frequency scale, words ranged from 7.22 to 13.67 (*M* = 10.15, *SD* = .88). In terms of concreteness, words ranged from 1.19 to 5.00 (*M* = 3.31, *SD* = 1.00).

#### Procedure

Participants were told that they would be presented with lists of words with each list containing 16 different words. Each word was shown one at a time, for 4 s each. After each list was presented, participants had 1 min to recall the words from just that list (i.e., not previous lists). Participants were also told that the words in each list would differ in point value and that they could score points by recalling words on the test. For example, if the word "apple" appeared on the list and they remembered "apple" during the test, then they would score points for recalling that word. Each participant’s goal was to maximize their score on each list. After each test, participants were told their score for that list but were not given feedback about specific items. This process was repeated for a total of eight study-test cycles.

Participants were instructed that the size of the font indicated the value of the word. Specifically, some participants (*n* = 55) were told that the bigger the font, the more points the word was worth if they recalled it, while other participants (*n* = 54) were told that the smaller the font, the more points the word was worth if they recalled it. On each list, four words appeared in 12-point font, four words appeared in 36-point font, four words appeared in 60-point font, and four words appeared in 84-point font. In the condition where the bigger the word, the greater the value (“big font = high value”), these font sizes corresponded to values of 2, 6, 10, and 14 points (the magnitude of these values directly maps onto the relative font sizes^4^). In the condition where the bigger the word, the lower the value (“big font = low value”), these font sizes corresponded to values of 14, 10, 6, and 2. The order of words within lists was randomized and words (which were never repeated) could appear on any list, in any order, and in any font size.

### Results

Memory selectivity can be computed via a selectivity index (see Castel et al., 2002) or memory performance can be modeled as a function of value. Modeling memory as a function of value controls for other variables included in the model, considers individual differences in value perception, and offers a more precise assessment of how value influences memory. Furthermore, employing item-level analyses allows every trial to be included for each participant, whereas the selectivity index represents a summary statistic across items in a list. Furthermore, there is an increasing consensus that researchers should avoid using ANOVAs (see Jaeger, 2008) and instead use item-level generalized logistic mixed-effect regression models. Thus, MLMs are recommended for this type of work (see Murphy, 2023).

**Fig. 3:**
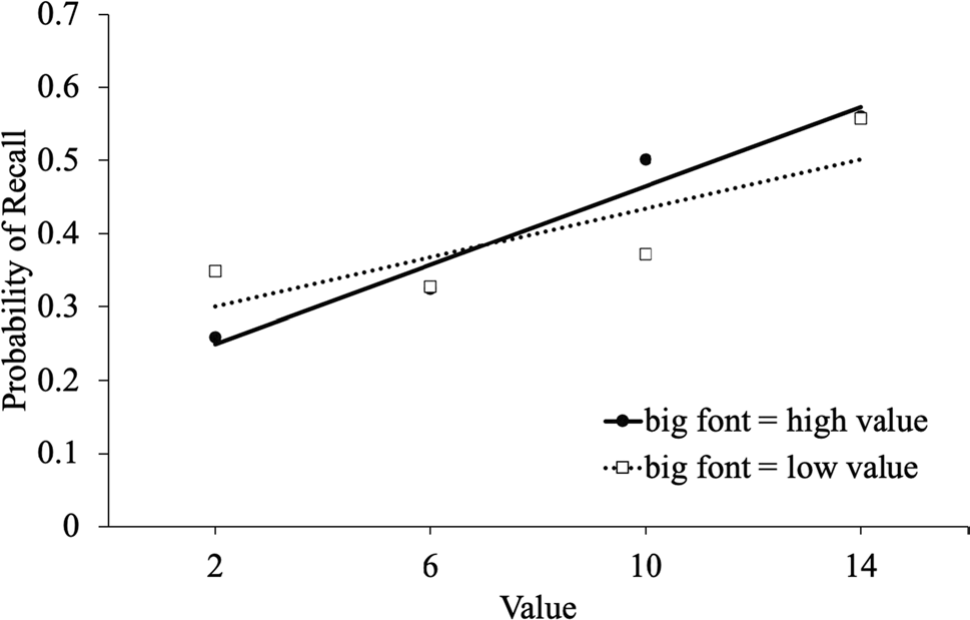
Linear trendlines for the probability of recall as a function of point value and scoring in Experiment 2

To examine memory selectivity (i.e., the propensity to better recall high-value words at the expense of low-value words; see Fig. 3), we conducted a logistic MLM^5^ with item-level recall modeled as a function of value, list, and scoring (big words worth higher values, small words worth higher values). Results revealed that value significantly predicted recall [e^B^ = 1.11, CI_95%_ = 1.10 – 1.11, *z* = 24.00, *p* < .001] such that high-value words were better recalled than low-value words. Participants recalled a similar proportion of words when they were told that big words were worth higher values (*M* = .41, *SD* = .13) as when they were told that small words were worth higher values (*M* = .40, *SD* = .14), [e^B^ = .98, CI_95%_ = .78 – 1.24, *z* = -.15, *p* = .878]. There was an effect of list [e^B^ = .98, CI_95%_ = .96 – .99, *z* = -2.98, *p* = .003] such that recall declined on later lists. Value interacted with scoring [e^B^ = .95, CI_95%_ = .94 – .97, *z* = -5.68, *p* < .001] (see Table 1 for descriptive statistics), and an analysis of the simple effects indicated that participants were more selective when bigger words were more valuable [e^B^ = 1.13, CI_95%_ = 1.12 – 1.14, *z* = 20.78, *p* < .001] than when smaller words were more valuable [e^B^ = 1.08, CI_95%_ = 1.07 – 1.09, *z* = 13.10, *p* < .001]. List interacted with value [e^B^ = 1.00, CI_95%_ = 1.00 – 1.01, *z* = 2.15, *p* = .032] such that participants were more selective on later lists. List did not interact with scoring [e^B^ = 1.00, CI_95%_ = .97 – 1.03, *z* = .10, *p* = .921], and there was not a three-way interaction between value, scoring, and list [e^B^ = 1.00, CI_95%_ = .99 – 1.01, *z* = -.57, *p* = .568].

**Table 1:** Descriptive statistics for the interaction between value/font size and scoring in Experiments 2 and 3. Standard deviations are in parentheses

	Size 12	Size 36	Size 60	Size 84
Experiments 2				
big font=high value	.26 (.17)	.32 (.16)	.50 (.18)	.56 (.18)
big font=low value	.66 (.27)	.37 (.20)	.33 (.21)	.35 (.22)
Experiments 3				
big font=high value	.42 (.21)	.42 (.17)	.45 (.17)	.49 (.17)
big font=low value	.49 (.16)	.44 (.16)	.46 (.17)	.50 (.17)

Beyond examining overall recall performance, we were also interested in how participants retrieved items – it is possible that participants used font size as a cue to recall the words. By employing a clustering recall assessment, we investigated whether participants organized their recall based on font size cues. To examine the recall patterns in relation to font size/value, as an exploratory analysis suggested by reviewers (not preregistered), we computed the Adjusted Ratio of Clustering (ARC; Roenker et al., 1971; Senkova & Otani, 2012). An ARC analysis provides a measure of how well participants’ patterns of recall align with the conceptual structure of the study materials. ARC is a reliable indicator of organization as it controls for variations in recall levels among participants or learning conditions, and quantifies the degree to which participants’ responses cluster based on predefined categories (here, the four font sizes/values). Scores on the ARC range from -1.0 to 1.0, with 0 indicating that the clustering observed in participants’ responses is no greater than what would be expected by chance alone and 1.0 indicating perfect clustering. In contrast, negative scores may indicate organizational patterns of recall that are not captured by conventional category clustering measures (for reviews of ARC and other clustering analyses, see Kahana et al., 2008; Murphy, 1979; Murphy & Puff, 1982; Pellegrino & Hubert, 1982).

To examine the degree to which participants clustered items from the same font size/value at recall, we calculated an ARC score for each list for each participant (Senkova & Otani, 2012). A one-sample *t*-test showed that, across conditions and lists, ARC scores (*M* = .10, *SD* = .31) were greater than 0 [*t*(107) = 3.52, *p* < .001, *d* = .34], indicating that participants tended to recall words of similar value/font size together. However, this tendency did not significantly differ between groups [*t*(106) = 1.89, *p* = .062, *d* = .36].

### Discussion

In Experiment 2, when participants were given explicit instructions as to how font size predicted point value, participants were more selective (i.e., better recall of high- relative to low-value words) when large words were worth more points than when small words were worth more points. This supports the notion that learners’ beliefs about font size involve large words being perceived as more valuable.

## Experiment 3

In Experiment 2, the value instructions were explicit (i.e., participants knew whether larger font sizes meant the word was worth more or fewer points). In Experiment 3, each word’s font size was either positively or negatively related to its point value (similar to Experiment 2), but the value scheme was not made explicit to participants. As such, participants had to determine the reward structure and then selectively encode the highest-valued words. For example, if a learner monitors their output, with feedback through multiple trials, they can deduce which types of items had the highest values. Thus, Experiment 3 sought to examine the role of inductive reasoning and implicit value inference in participants’ selective memory processes.

By investigating participants’ ability to implicitly determine the value structure based on font size, Experiment 3 provides deeper insights into learners’ implicit beliefs about font size as an indicator of importance. Experiment 1 demonstrated that participants rated words in large font as more important while Experiment 2 showed that participants were more selective when font size was positively related to value. Thus, in Experiment 3, we hypothesized that participants would be quicker to learn the value scheme when words in large font were associated with greater point values, as this aligns with participants’ beliefs about font size as an indicator of importance with the value structure.

### Method

#### Participants

Participants were 107 undergraduate students (*M*_*age*_ = 20.47 years, *SD*_*age*_ = 2.04 years). No participants were excluded for cheating.

#### Materials and procedure

The materials and procedure in Experiment 3 were similar to Experiment 2. For some participants (*n* = 54), the bigger the font the more points the word was worth if they recalled it; for other participants (*n* = 53), the smaller the font the more points the word was worth if they recalled it. Of note, participants did not receive explicit value instructions.

### Results

**Fig. 4:**
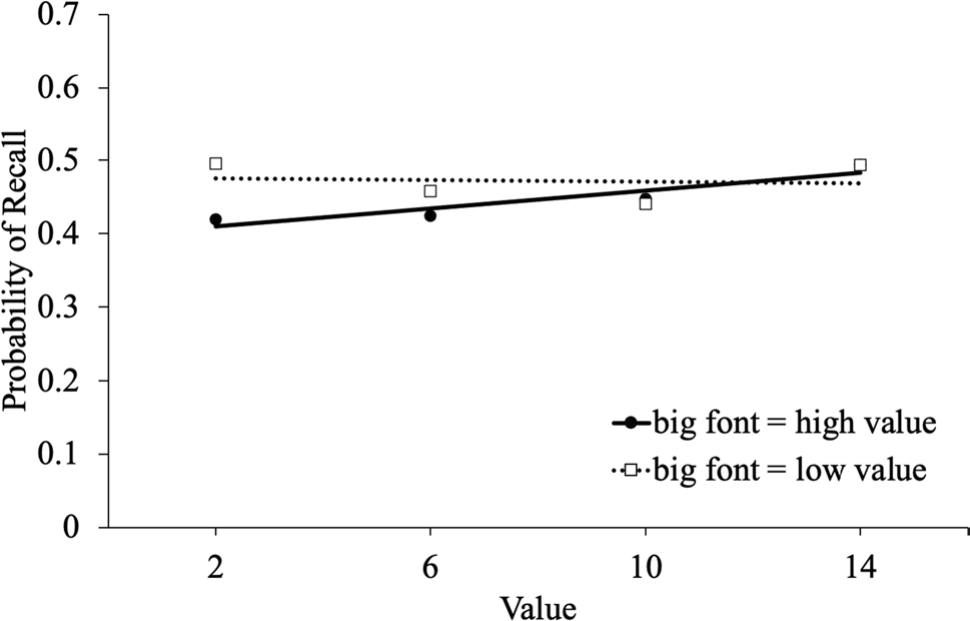
Linear trendlines for the probability of recall as a function of point value and scoring in Experiment 3

To examine memory selectivity (see Fig. 4), we conducted a logistic MLM^6^ with item-level recall modeled as a function of value, list, and scoring (big words worth higher values, small words worth higher values). Results revealed that value significantly predicted recall [e^B^ = 1.01, CI_95%_ = 1.00 – 1.02, *z* = 3.07, *p* = .002], such that high-value words were better recalled than low-value words. However, participants recalled a similar proportion of words when big words were worth higher values (*M* = .45, *SD* = .14) as when small words were worth higher values (*M* = .47, *SD* = .14), [e^B^ = 1.13, CI_95%_ = .89 – 1.44, *z* = 1.03, *p* = .302]. There was not an effect of list [e^B^ = 1.01, CI_95%_ = .99 – 1.02, *z* = .95, *p* = .342]. Value interacted with scoring [e^B^ = .97, CI_95%_ = .96 – .99, *z* = -3.71, *p* < .001] (see Table 1 for descriptive statistics), and an analysis of the simple effects indicated that participants were only selective when bigger words were more valuable [e^B^ = 1.03, CI_95%_ = 1.02 – 1.04, *z* = 4.79, *p* < .001]. However, participants were not selective when smaller words were more valuable [e^B^ = 1.00, CI_95%_ = .99 – 1.01, *z* = -.45, *p* = .651]. List interacted with scoring [e^B^ = 1.03, CI_95%_ = 1.00 – 1.06, *z* = 1.99, *p* = .047], and an analysis of the simple effects indicated that recall increased on later lists for participants in the condition where bigger words were less valuable [e^B^ = 1.02, CI_95%_ = 1.00 – 1.05, *z* = 2.08, *p* = .038]; recall did not change throughout the task for participants in the condition where bigger words were more valuable [e^B^ = .99, CI_95%_ = .97 – 1.01, *z* = -.74, *p* = .462]. List did not interact with value [e^B^ = 1.00, CI_95%_ = 1.00 – 1.00, *z* = .47, *p* = .637], and there was not a three-way interaction between value, scoring, and list [e^B^ = 1.00, CI_95%_ = 1.00 – 1.01, *z* = .63, *p* = .529].

Inspection of Fig. 4 suggests that a quadratic pattern may be present when bigger words were worth fewer points.^7^ Indeed, an exploratory quadratic MLM with value predicting recall for participants where bigger words were worth fewer points indicated that there was a significant quadratic trend [e^B^ = 1.01, CI_95%_ = 1.00 – 1.01, *z* = 3.87, *p* < .001], but this was not the case for participants when bigger words were worth more points [e^B^ = 1.00, CI_95%_ = 1.00 – 1.01, *z* = 1.80, *p* = .072]. Thus, in the condition where larger fonts corresponded to lower values, the data suggest that participants exhibited better recall for words at both extremes of the point spectrum – those presented in large font with lower associated value (2 points) and those in small font with higher value (14 points). Thus, participants may initially adhere to their pre-existing beliefs by recalling words in a large font more effectively, but also refine their understanding of the reward system by improving their recall of high-value words presented in a small font.

We again calculated an ARC score for each list for each participant. A one-sample *t*-test demonstrated that, across conditions and lists, ARC scores (*M* = .05, *SD* = .18) were greater than 0 [*t*(106) = 3.04, *p* = .003, *d* = .30], indicating that participants tended to recall words of similar value/font size together. However, this tendency did not significantly differ between groups [*t*(104) = -.73, *p* = .446, *d* = -.14].

### Discussion

In Experiment 3, when learners had to learn the value structure (as it relates to font size) without explicit instruction (i.e., implicitly; participants were not explicitly given the scoring scheme), they were only selective when large words were worth more points – participants were not selective when small words were worth more points. In this task, the only way to determine the value structure was for participants to monitor their output on early lists (e.g., if lists where participants recalled mostly large words yielded higher scores than lists where they recalled mostly smaller words, this monitoring of output and feedback would reveal the value structure). The fact that participants were sensitive to value suggests that they used this strategy, but this pattern was obtained only when the value structure fit participants’ beliefs (i.e., higher values correspond to words in bigger fonts). Thus, learners appear to view font size as an indicator of importance or value.

## General discussion

We are often presented with large amounts of information to remember and, in many cases, the font size of information may be indicative of its importance (see Luna et al., 2019). For example, important vocabulary terms are often bolded or highlighted, and newspaper headlines are usually printed in large font with bigger stories often appearing in bigger font sizes (see Ball et al., 2014, for work on memory for bolded information; see Maxwell et al., 2021, for work on different font types). In the current experiments, we were interested in how learners perceive the value of information in different font sizes as one’s beliefs about larger fonts may bias and influence value-based memory processes.

In Experiment 1, we presented participants with unrelated and related lists of to-be-remembered words in either small or large font. Additionally, we asked participants to judge the importance of remembering each word (JOI). By assessing JOIs, we could determine whether people explicitly regard words in larger fonts as more important to remember than words in smaller fonts. Results revealed that when the words in a list were unrelated, participants rated words in large font as more important to remember (consistent with Luna et al., 2019). However, when the word lists contained broad themes that were schematically related to a goal (i.e., items to pack for a vacation), font size did not significantly impact JOIs. Thus, the present study indicates that font size may be a cue indicating the importance of some to-be-remembered information. However, when this information varies in subjective value and there is a goal to remember certain items, the importance of remembering can greatly reduce any effects of font size (see also Murphy et al., 2022, for an example of value superseding font size). Although we examined this using a list of words related to a goal where differences in font size were clearly noticeable, it would be informative to further examine situations in which changes in font size are either subtler/less noticeable or exist on a greater continuum (see Halamish, 2018), when font size is consistent across sentences/text (such as an entire book that is presented in smaller or larger print; see Katzir et al., 2013), or when reading medication information that may be very important but is presented in a smaller font (side effects of a medication; see Hargis & Castel, 2018).

In Experiments 2 and 3, we investigated how a learner’s belief that font size is indicative of importance relates to their ability to selectively encode and recall valuable information. Specifically, we presented learners with words in various font sizes whereby larger fonts either corresponded to greater point values or smaller point values (between subjects). In Experiment 2, participants were made explicitly aware of this value structure, and results revealed that when larger fonts corresponded with greater point values, participants were better able to selectively encode and recall high-value words relative to low-value words, suggesting that a congruency exists whereby larger font words are perceived as more important.

In Experiment 3, participants were not explicitly told the value structure – it was up to them to determine which words were valuable. Results revealed that when bigger words were more valuable, learners were better able to encode and recall these items relative to smaller, lower-valued words, illustrating selective memory. However, when smaller words were more valuable, learners were unable to discern the value structure and engage in selective memory. Together, Experiments 2 and 3 provide further evidence that, even after some task experience, learners’ beliefs (Mueller et al., 2014; Undorf & Zimdahl, 2019) drive the font size effect (greater expected memory for large relative to small words; Ball et al., 2014; Besken & Mulligan, 2013; Blake & Castel, 2018; Halamish, 2018; Kornell et al., 2011; Luna et al., 2018; Mueller & Dunlosky, 2016; Price et al., 2016; Rhodes & Castel, 2008), such that font size is seen as a proxy of importance (see Luna et al., 2019). However, the present study did not employ a pure measure of beliefs given that participants also have the experience of engaging in encoding, suggesting an agenda for future work.

When valuable words are presented in a smaller font (which led to reduced selectivity in Experiment 2 and an absence of selectivity in Experiment 3), participants may not engage in differential rehearsal as the perception of these words does not match with pre-existing beliefs about how the reward structure should be associated with font size. In terms of a cue-utilization framework (Koriat, 1997), learners may be placing too much weight on the influence of intrinsic qualities of words, including font size. Specifically, learners may think that these factors can influence memorability and struggle to override these beliefs when engaging in selective memory.

Experiment 3 illustrated that participants can learn to determine the value of information even if that value is not made explicit. In some prior work (e.g., Silaj et al., 2023), learners ascertained the value of to-be-remembered information by understanding its place within the schematic structure of other items in a list (e.g., learning that examples of fish are worth more points and thus more valuable to remember relative to examples of furniture), although it is unclear if this occurs via an explicit awareness of varying levels of importance being associated with different categories of items. Here, we demonstrated the influential role of people’s beliefs about font size in guiding JOIs and subsequent memory. Although font size may not directly affect recall (e.g., Mueller et al., 2014), our results suggest that individuals default to perceiving information presented in larger font as more important to remember. This tendency to associate larger font size with greater importance could influence memory processes and guide individuals’ attention towards prioritizing information displayed in a larger font. Furthermore, information in a smaller font may not be regarded as important even when (as in the present work) the reward structure is set up such that this information is valuable. While people’s beliefs are often shaped and reinforced by experience (e.g., larger headlines for important news), there may also be instances where one needs to update these beliefs (e.g., important or useful details in the fine print/footnotes).

In addition to examining overall recall performance, we sought to explore how participants retrieved items and whether they used font size as a cue during recall. As such, we employed a clustering recall assessment (Adjusted Ratio of Clustering (ARC); Roenker et al., 1971; Senkova & Otani, 2012), which enabled us to assess whether participants organized their recall based on font size/value cues. ARC scores in Experiments 2 and 3 indicated that participants may have used font size or value as a cue to recall the words, as evidenced by the clustering patterns observed in their recall responses. Interestingly, clustering did not differ depending on whether larger font sizes were associated with high values or low values. These findings suggest that participants may have relied on font size or value as a general cue for recall, irrespective of the specific value assigned to each font size. This highlights the importance of considering the influence of font size and value as potential cues in memory retrieval processes.

In Experiment 1, the approach of explicitly asking participants to rate the importance of words presented in different font sizes offers direct insight into participants’ explicit beliefs and judgments regarding font size and importance. In Experiments 2 and 3, we employed a more implicit approach to measure the impact of font size on importance. By manipulating the point values associated with different font sizes (and in Experiment 3, without explicitly informing participants about the value structure), we aimed to examine participants’ implicit beliefs and expectations regarding the relationship between font size and importance. This approach allowed us to examine whether participants could discern the value structure and selectively encode and recall high-value words based on font size alone, without explicit instructions. Furthermore, using an implicit approach helps us explore the underlying cognitive processes and automatic associations that may influence the perception of font size and importance.

Future work may benefit from further examining how a learner’s beliefs drive subsequent selective memory processes, and whether they are explicitly aware of these beliefs and processes. One potential approach for investigating learners’ explicit awareness of their beliefs and processes is to include a metacognitive component in the experimental design. For example, after participants have completed the memory task, they could be asked to reflect on their strategies for selecting and remembering high-value words. Researchers can employ think-aloud protocols or post-task interviews to gain insights into participants’ thought processes, including their awareness of font size as a cue for importance and their deliberate strategies for encoding and recalling valuable information. Future work could also benefit from larger sample sizes and/or incorporating more learning trials as the intricacy of our models made analyzing the power of the current experiments difficult. Additionally, we examined situations in which font size was not necessarily diagnostic of recall or importance but could influence JOIs and subsequent memory. However, it may be useful to further examine how font size could capture attention and guide importance ratings (such as with headlines or warning labels). In such cases, there is a stronger correlation between font size and the significance of information, potentially leading to higher JOIs and influencing recall more directly (as also noted by Luna et al., 2019).

We note two limitations of the current work. First, word frequency differed between related and unrelated words in Experiment 1 (see Open Science Framework (OSF) for analyses from Experiment 1 that control for word frequency). While word frequency can have a positive correlation with recall (Hall, 1954), it is important to note that our primary focus in Experiment 1 was to examine the relative differences in JOIs between related and unrelated words, which should not be directly influenced by frequency (especially given the between-subjects design). Although empirical studies have shown that more frequent items tend to receive higher JOLs (e.g., Benjamin, 2003), JOLs reflect predictions of future memory performance while the JOIs pertain to participants’ judgments of the importance of remembering each word. Given this distinction, it is possible that the relationship between word frequency and JOIs may differ from that observed in JOL studies. The primary focus of our study was to investigate the influence of font size and relatedness on JOIs and subsequent memory performance, rather than directly examining the effects of word frequency. Nonetheless, although font size was not confounded with status as a related or unrelated word, we note that frequency may have had some influence on JOI judgments, suggesting some caution in interpreting the results.

Second, we also note that several of our effect size estimates were small, underscoring the complexity of memory processes and indicating that the way individuals perceive and recall information is likely a multifaceted phenomenon, influenced by a constellation of factors beyond font size and perceived importance. Relatedly, our MLMs and ANOVAs did not always produce identical patterns of results (reported on the OSF). The MLM approach was most appropriate for our study design due to its ability to handle nested data and variability among participants. However, the dependence of our results on the statistical method used does call into question the robustness of our findings. Future work could further examine how beliefs influence memory selectivity using more study-test trials, bigger sample sizes, as well as different forms of memory assessment.

The present study offers valuable insights into how individuals perceive and learn information of varying importance presented in different font sizes. Specifically, these findings have implications for real-life scenarios whereby the importance of information may not always align with its font size. For example, certain critical details such as footnotes, fine print in contracts, or training manuals may be presented in smaller font, despite their significance. This has implications for individuals with vision or hearing impairments, particularly older adults, who may judge information as less important if it is difficult to perceive. Older adults may benefit when information intensity is a valid predictor of information importance and/or when memory aids could be provided that identify what information is important based on physical and sensory properties (e.g., effectively offloading information may be facilitated by presenting items on a list in order of importance, where font size also contributes to the perceived level of importance; see Murphy & Castel, 2023).

There could be educational and training implications as well, such as when learning a new language, ensuring that frequently used translations are presented in larger font, especially if these are important to know for future use on a trip (Murphy et al., 2023a). However, students could sometimes be misled by larger fonts being more fluent and perceived as more important, and may have overconfidence if larger-font information is not learned well. Finally, when reading information on medication inserts and instructions, one may perceive that a long list of mild and serious side effects of a medication may not be considered as important or frequent if presented in a smaller font (see also Hargis & Castel, 2018). Thus, future research could examine the implications of how font size can influence JOIs in various settings and other populations.

In sum, although font size is often not very indicative of later memory (e.g., Chang & Brainerd, 2022), learners may judge words in large font as more likely to be remembered because words in large font are often associated with greater importance (see Luna et al., 2019). Specifically, when words in a list are unrelated and learners are devoid of cues to differentiate the importance of to-be-learned material, a word’s font size may be predictive of its importance. However, when to-be-remembered information varies in subjective value and there is a goal of remembering certain items, font size may be less diagnostic of an item’s importance, and the item’s subjective importance drives memory. The present work shows how attitudes about larger font sizes guide value-based beliefs about importance and memory, and when the value or importance of information is consistent with a learner’s beliefs, they may be better able to engage in selective memory.

## Appendix

**Table 2:** Stimuli used in Experiment 1

Going camping	Going on vacation	Child’s party	Going to class	Making lasagna	Going on a picnic
ax	backpack	ballons	calculator	basil	apples
boots	batteries	cake	chapstick	butter	basket
cards	book	clown	coffee	eggs	blanket
chair	camera	confetti	eraser	flour	bread
clock	hat	cooler	flashdrive	garlic	candles
compass	medication	flowers	glasses	milk	cheese
cups	money	forks	highlighter	mushrooms	chicken
lantern	passport	fruit	iPad	noodles	cookies
map	razor	games	keys	onions	frisbee
marshmallows	sandals	goodybags	kleenex	oregano	jacket
matches	shampoo	invitations	laptop	oven	knife
pants	shirt	juice	notebook	pan	napkins
pillow	shorts	music	paper	parmesan	plates
shovel	snorkel	pens	pencil	parsley	radio
soap	socks	pinata	phone	pepper	salad
tarp	speaker	pizza	ruler	ricotta	sandwich
tent	sunscreen	present	snack	salt	soda
water	swimsuit	pretzels	sweater	sauce	thermos
whistle	toothbrush	streamers	wallet	spinach	watermelon
wood	towel	tables	watch	tomatoes	Ziplocks

## Footnotes

1 We note that, in their meta-analysis, Chang and Brainerd (2022) reported a reliable effect of font size on recall. However, the effect was quite small (*g* = .05) and unlikely to be detectable in experiments without extraordinary power (e.g., via a paired *t*-test, one would need to test 3,142 participants to have 80% power to detect the effect, assuming a two-tailed test and an alpha level of .05).

2 The word length difference was unintentional and not a deliberate design choice. In terms of word frequency, an independent-samples *t*-test revealed that unrelated words had a significantly higher frequency than related words [*t*(224) = 6.35, *p* < .001, *d* = .85]. While frequency can be positively correlated with recall (Hall, 1954), our primary interest in Experiment 1 was to examine the relative differences in JOIs between related and unrelated words, which should not be directly affected by frequency (especially given the between-subjects design). However, we acknowledge that the frequency difference between related and unrelated words could potentially introduce a confound in the memory test. See Open Science Framework link for analyses controlling for differences in frequency statistically. There were few differences in the results when controlling for frequency.

3 The narrow confidence intervals (CIs) observed for some significant results, such as this effect size of 1.01 with CI 1.01-1.01, indicate a precise estimation of the effect size. In these cases, the point estimate is very close to 1, suggesting a minimal but statistically significant effect. The narrow CIs reflect a high level of certainty in the estimated effect size, as the interval is tightly constrained around the point estimate. However, wider CIs observed for non-significant results indicate greater uncertainty regarding the estimated effect size. In these instances, the wider range of the CI reflects the possibility of a wider range of effect sizes that could potentially exist in the population.

4 Each increase of 1 point in value corresponded to a change in font size by 6. This means that there was a consistent increment of 6 points in font size for every 1 unit increase in value. For example, in the condition where "big font = high value," the smallest font size of 12-point corresponded to a value of 2. The next highest value of 6 points was thus presented in 36-point font. By applying this rule of a 6-point change in font size for every 1-unit change in value, we were able to establish a systematic and consistent relationship between the font sizes and the assigned values.

5 See Open Science Framework link for a similar analysis using an ANOVA. All of the significant effects in the MLM were significant in the ANOVA.

6 See Open Science Framework link for a similar analysis using an ANOVA. Most of the significant effects in the MLM were significant in the ANOVA. However, the interaction between size/value and scoring did not reach statistical significance.

7 We thank a reviewer for identifying this possibility.
